# Harmonizing Logical Observation Identifiers Names and Codes (LOINC) Codes and Units in Real-World Oncology Data: Method Development and Evaluation

**DOI:** 10.2196/81254

**Published:** 2026-03-09

**Authors:** Parvati Naliyatthaliyazchayil, Travis Stenerson

**Affiliations:** 1 ConcertAI, LLC Cambridge, MA United States

**Keywords:** real-world data, laboratory data, harmonization, standardization, oncology, health care system, electronic health record, multisourced data, structured data, mapping, Fast Healthcare Interoperability Resources, FHIR, Logical Observation Identifiers Names and Codes, LOINC, Systematized Nomenclature of Medicine, SNOMED, unit conversion

## Abstract

**Background:**

The expanding use of multisource real-world electronic health record (EHR) and claims data offers major opportunities for research, drug discovery, and clinical decision support. While standards such as Logical Observation Identifiers Names and Codes (LOINC) can ensure semantic interoperability for laboratory observations, clinical documents, and other clinical terms, properly assigning these concepts remains a challenge. Studies show that 6% to 19% of laboratory tests cannot be accurately mapped to LOINC. Existing systems try to address this challenge but often depend on source data strings and other input features that may be absent, null, or incorrect. This underscores the need for a scalable approach to correct LOINC code assignments, standardize units, and ensure data integrity across multisource laboratory data.

**Objective:**

This paper presents a universally applicable framework that identifies and corrects observable errors in quantitative laboratory results coded to LOINC and the Systematized Nomenclature of Medicine for the unit of measure without relying on raw source data strings. The process seeks to improve the accuracy, conformance, consistency, and completeness of laboratory data while maintaining complete provenance.

**Methods:**

The proposed framework uses a 2-step process. First, LOINC codes are corrected using the associated unit of measure. Second, units are adjusted or populated to match a preferred unit for that LOINC code. In both steps, the quantitative result is checked against a predefined acceptable range to determine validity. The process is driven by 3 knowledge tables. The framework is applied to datasets derived from the ConcertAI database of approximately 10 million patients with cancer, evaluating improvements in LOINC code–unit conformance and unit completeness. Analyses are performed on 4 independently LOINC-coded datasets: the full ConcertAI dataset and 3 high-volume diverse subsets grouped by data source or EHR vendor.

**Results:**

A total of 428 LOINC codes were observed across 6.34 billion records in the ConcertAI database. All 4 datasets were processed using the proposed framework. Before applying the framework, 73.1% (4,634,610,173/6,337,101,453) of records in the ConcertAI dataset had correctly assigned units based on the laboratory reasonable range table; after application, this increased to 99.7% (6,322,375,200/6,341,230,213). Similar improvements were observed across the 3 EHR-specific datasets, increasing from 78.5% (691,315,390/880,250,137) to 99.8% (879,626,472/881,157,852; source 1), 71.4% (2,132,455,936/2,985,465,124) to 99.8% (2,982,319,644/2,988,173,959; source 2), and 63.3% (2,936,710,502/4,640,432,294) to 99.6% (4,618,714,114/4,638,862,412; source 3). Unit completeness also improved substantially, increasing from 92.7% (5,879,071,858/6,341,230,213) to 99.8% (6,331,923,060/6,341,230,213) in the ConcertAI dataset and from 92.5% (814,867,241/881,157,852) to 100% (880,816,133/881,157,852), 94.4% (2,822,107,252/2,988,173,959) to 99.9% (2,986,624,027/2,988,173,959), and 91.7% (4,254,054,966/4,638,862,412) to 99.8% (4,632,935,919/4,638,862,412) in sources 1 to 3, respectively.

**Conclusions:**

Laboratory data quality is crucial in oncology systems for therapy selection, monitoring, and disease progression assessment. This proposed solution is a first-of-its-kind, system-agnostic, and scalable normalization process that addresses key gaps in laboratory data quality across multiple dimensions.

## Introduction

### Background: Real-World Data in Health Care

Multisourced, comprehensive patient health data have seen a recent rise in prominence and utility in supporting research, drug discovery, pharmacovigilance, and clinical decision support [1]. These real-world data (RWD) are collected for individual patient care and reimbursement in often unconnected systems such as electronic health records (EHRs) and claims clearing houses [2]. Secondary use of RWD holds immense potential for improving health due to its volume, variety, and ubiquity [3].

The different source systems producing RWD bring individual challenges and opportunities, and it is often necessary to stitch together disparate sources to cover a complete patient journey [3]. For instance, medical claims may contain procedures and tests performed on a patient and their associated costs, but the results of those tests are only present in the EHR [4].

### Data Quality and Semantic Interoperability Challenges

The path to a usable RWD dataset is fraught with challenges [2]. Errors can arise from any system or actor that generates, transmits, transforms, or persists the data during its lifetime [5]. Errors can be discernible from patterns observed in the data or can be hidden from downstream users when information is lost [6]. Information loss can result in incorrectness or missingness of variables, records, tables, or complete data sources [7]. Monitoring and minimizing information loss is a critical requirement of an RWD pipeline.

Semantic interoperability (SI), or the ability to ensure the same meaning and interpretation of data shared between systems [8], is a well-established and ongoing challenge in RWD [5]. It can manifest as redundant representations of the same concept, such as one EHR using the *International Classification of Diseases, 10th Revision, Clinical Modification*, and another using the Systematized Nomenclature of Medicine (SNOMED) to represent the same disease. Use of data standards and common ontologies can alleviate SI challenges but can also introduce errors if used incorrectly [5], which can commonly occur when a concept is assigned to a string by either a human or machine [9]. As health care data systems continue to evolve, the importance of SI is underscored by the increasing adoption of standards such as Fast Healthcare Interoperability Resources (FHIR) [10] with its associated code system bindings supported by the Food and Drug Administration, which are designed to ensure consistent data interpretation and improve interoperability across systems. Even FHIR-based representations may fall short in fully addressing semantic mismatches, particularly for complex domains such as laboratory data, in which variability in coding, units, and local conventions persists despite standardization efforts [11].

### Laboratory Data and Logical Observation Identifiers Names and Codes Mapping Challenges

Laboratory result data are both valuable and particularly challenging in RWD [9]. To address SI, laboratory data primarily rely on codes from the Logical Observation Identifiers Names and Codes (LOINC) code system [12,13]. LOINC is the dominant global coding system for laboratory concepts but also extends to a broader range of clinical terms, including clinical questions and documents [12-15]. LOINC consists of concepts that have 6 attributes each. These include the analyte tested, how it is observed, the duration of observation, and the sample type that the observation is performed on [13]. Precoordinating that much information into a single concept makes it convenient for transmission by reducing the potential for misinterpretation but also makes it difficult to use when mapping to LOINC [14,15]. The complete information for LOINC mapping is rarely present in a single string, column, or record [15]. Relevant information may be spread across one or more fields in a record or laboratory information system, such as the observation’s display string (eg, glucose), the data type of the result (numeric or categorical), the unit of measure (mass over volume or molarity), the associated order name or panel name (complete blood count or urinalysis), the associated sample (urine or blood), the device or equipment used to make the observation (urine dipstick or continuous glucose monitor), or other observations that occurred as part of the same panel of tests (Is the patient fasting?). Despite the abundance of resources, accurately translating these fields to the correct LOINC code remains a challenge.

This semantic and translational complexity means that laboratory results in RWD can frequently present with errors introduced during terminology binding to LOINC [16]. Prior studies have shown that LOINC codes could not be assigned for 6% to 19% of laboratory tests due to incomplete or incorrect information [17]. Systems have been proposed to reduce this error by coding via automated means in RWD [9,16,18] and clinical trial data [19]. These algorithms rely on source data strings and other input features that may be absent, null, or observably incorrect.

### Unit of Measure and Normalization Challenges

The diversity of information required for LOINC mapping increases the likelihood of mapping difficulty [20] and significant information loss. If a result’s unit of measure is absent or recorded in the wrong place or the associated panel name, order name, or sample type is missing from the data, it can mean insufficient information to properly assign the LOINC code [15]. Contextually appropriate filling in of absent units of measure has been shown to be effective in a system that extracts logical expressions from clinical trial inclusion criteria pertaining to quantitative laboratory results but not within RWD [21].

A laboratory result may present with a correct concept code, but it is possible to represent the same result using multiple scales (g or mg), and certain units are used interchangeably between sources (mg per day or mg per 24 hours). Unit normalization is necessary to ensure comparable results during data analysis and aggregation, and the variety of representation, the missingness, and the level of incorrectness can all lead to loss of utility of RWD. Even with a correct LOINC code, missing or inconsistent units have been shown to affect as much as 14% of laboratory records in some systems [22,23]. Mechanisms to normalize synonymous units exist, but these techniques do not handle situations in which the unit is incomplete, incorrect, or completely absent [24].

### Proposed Framework and Objective: Correcting Quantitative Laboratory Results

In this paper, we present a framework to identify and correct observable errors in quantitative laboratory results that have already been coded to LOINC for the observation and SNOMED for the unit of measure without reliance on source data strings. This 2-step process first adjusts the LOINC code by using information contained in the associated unit of measure. Next, we use the quantitative result to inform whether we can safely populate a missing unit of measure or correct an erroneous unit of measure. Finally, all synonymous or related units are normalized to a single preselected unit for each LOINC code. We characterize the prevalence of these errors and then use this framework to correct laboratory results in a multisource RWD pipeline of oncology patient data from EHRs, laboratory information systems, and medical claims.

## Methods

This section outlines the data collection and study design used to develop this framework.

### Data Collection

Laboratory result data were extracted from the ConcertAI database, a US-based, deidentified, patient-level dataset from approximately 10 million patients with cancer aged ≥18 years during the period from 2015 to 2025. The data used in this study were sourced from EHRs, including oncology practices, hospitals, and academic medical centers. To evaluate the framework, we analyzed 4 laboratory datasets: the full ConcertAI database and 3 subsets corresponding to the individual EHR sources (represented as source 1, source 2, and source 3) that transmitted data to ConcertAI. These 3 subsets were selected because they were the top contributors of laboratory data by volume and represented a broad diversity of laboratory tests. Specifically, the ConcertAI database accounted for 6.34 billion records, source 1 included 880 million records, source 2 included 2.98 billion records, and source 3 included 4.64 billion records.

Data were contributed by multiple clinical sites and laboratories across the United States, representing a diverse group of care settings. Each dataset was standardized using LOINC codes for laboratory observations and SNOMED codes for units of measure. For records from the full ConcertAI database, LOINC codification was performed using ConcertAI’s internal system; for EHR or data source–derived subsets, the LOINC codes that were delivered with those data were used. Only records with a numeric value were used in this study. All datasets used ConcertAI’s unit codification that maps unit strings to SNOMED.

### System Design

The laboratory correction framework was applied after standardization to LOINC and SNOMED. The laboratory correction system relies on 3 knowledge tables that have been manually created after careful examination of the ConcertAI laboratory data, as outlined below. Their role in supporting the logical framework is discussed later in this section. Below, we provide the formal definitions of these tables, including their constituent fields and primary keys. The procedures used to create each table and the column definitions are documented in Multimedia Appendix 1.

The LOINC conversion map (LCM) relates a LOINC concept and unit concept that are incongruous to a new LOINC code that is compatible with the unit of measure. The table structure incorporates the following computable fields: “old_loinc_code,” “old_loinc_display,” “old_loinc_system,” “unit_code,” “unit_display,” “unit_system,” “new_loinc_code,” “new_loinc_display,” and “new_loinc_system.” The primary key for the table is defined as the composite of “old_loinc_code” and “unit_code.” Examples are shown in Table 1, which has been formatted for readability. The complete computable version is available in Multimedia Appendix 1 and in the accompanying GitHub repository to support transparency and reproducibility.

**Table 1:** Logical Observation Identifiers Names and Codes (LOINC) conversion map.

LOINC code	Unit (SNOMED^a^)	Corrected LOINC code
Platelets (#/volume) in blood (26515-7)	fL (258775009)	Platelet (entitic mean volume) in blood (28542-9)
Glucose (mass/volume) in blood (26515-7)	mEq/L (258865000)	Glucose (moles/volume) in blood (15074-8)
Glucose (mass/volume) in blood (26515-7)	mmol/L (258813002)	Glucose (moles/volume) in blood (15074-8)
Kappa light chains (mass/volume) in serum or plasma (11050-2)	Percentage (118582008)	Kappa lymphocytes/lymphocytes in blood (17096-9)

^a^SNOMED: Systematized Nomenclature of Medicine.

The reasonable range map defines metadata about a single LOINC code, including its assigned correct unit of measure, a minimum and maximum value that the laboratory test can reasonably take, and the mean and median of records with that LOINC code and unit across all 4 datasets. The correct unit of measure for each LOINC code was determined using the “example_unit” supplied in the official LOINC distribution. These units were mapped to the corresponding SNOMED codes. For LOINC codes that listed more than one example unit, we examined the empirical distribution of units observed in our dataset and selected the unit with the highest frequency. This made the choice of the correct unit data driven and ensured that the process could be consistently reproduced in any system. If Unified Code for Units of Measure (UCUM) units are preferred, LOINC also provides “example_ucum_units,” which can be incorporated similarly. Minimum and maximum reasonable values for each LOINC code were defined by estimating the empirical distribution of the data through quantiles of 0.005, 0.025, 0.16, 0.5 (median), 0.84, 0.975, and 0.995. The range was set at or near the highest and lowest of those quantiles, providing broad yet data-anchored thresholds for plausibility checking.

This knowledge table includes the following computable fields: “loinc_code, loinc_name,” “unit_code,” “unit_name,” “min_reasonable,” “max_reasonable,” “mean,” and “median.” The primary key for this table is the “loinc_code.” A readability-optimized version is shown in Table 2, whereas the complete computable version is provided in Multimedia Appendix 1 and the GitHub repository.

**Table 2:** Reasonable range map.

LOINC^a^ code	Unit (SNOMED^b^)	Reasonable range	Median	Mean
Platelets (#/volume) in blood (26515-7)	×10(3)/mcL (1287856009)	1-800	223.5	215.0
Glucose (mass/volume) in blood (2339-0)	mg/dL (258797006)	40-400	138.8	121
Kappa light chains (mass/volume) in serum or plasma (11050-2)	mg/L (258796002)	0.1-5000	68.7	25.9

^a^LOINC: Logical Observation Identifiers Names and Codes.

^b^SNOMED: Systematized Nomenclature of Medicine.

The unit multiplier map relates a correct unit to a synonymous, convertible, or incorrect unit along with a multiplier value, an additive scalar value, and a description of the relationship between the 2 units (eg, incorrect, synonym, or convertible). The scalar value is only used for temperature conversions. An empty “incorrect unit” represents a null unit field and is used to populate absent units. This knowledge table includes the following computable fields: “incorrect_unit_code,” “incorrect_unit_name,” “correct_unit_code,” “correct_unit_name,” “multiplier,” “scalar_constant,” and “multiplier_type.” The primary key is the composite of “incorrect_unit_code,” “correct_unit_code,” and “multiplier_type.” A readability-optimized version is shown in Table 3, with the full computable version available in Multimedia Appendix 1 and the GitHub repository.

**Table 3:** Unit multiplier map.

Incorrect unit (SNOMED^a^)	Correct unit (SNOMED)	Multiplier	Scalar	Multiplier type (description)
<null>	×10(3)/mcL (1287856009)	1	0	“nullfill” (unit absent)
10*9/L (277288007)	×10(3)/mcL (1287856009)	1	0	“synonym”
cells/µL (258878000)	×10(3)/mcL (1287856009)	0.001	0	“unit_conversion” (convertible unit)
cells/µL (258878000)	×10(3)/mcL (1287856009)	1	0	“unit_typo” (known unit error)
g/dL (258795003)	mg/dL (258797006)	1000	0	“unit_conversion” (convertible unit)
g/dL (258795003)	mg/dL (258797006)	1	0	“unit_typo” (known unit error)
g/L (258794004)	mg/dL (258797006)	1	0	“unit_conversion” (convertible unit)

^a^SNOMED: Systematized Nomenclature of Medicine.

**Figure 1:**
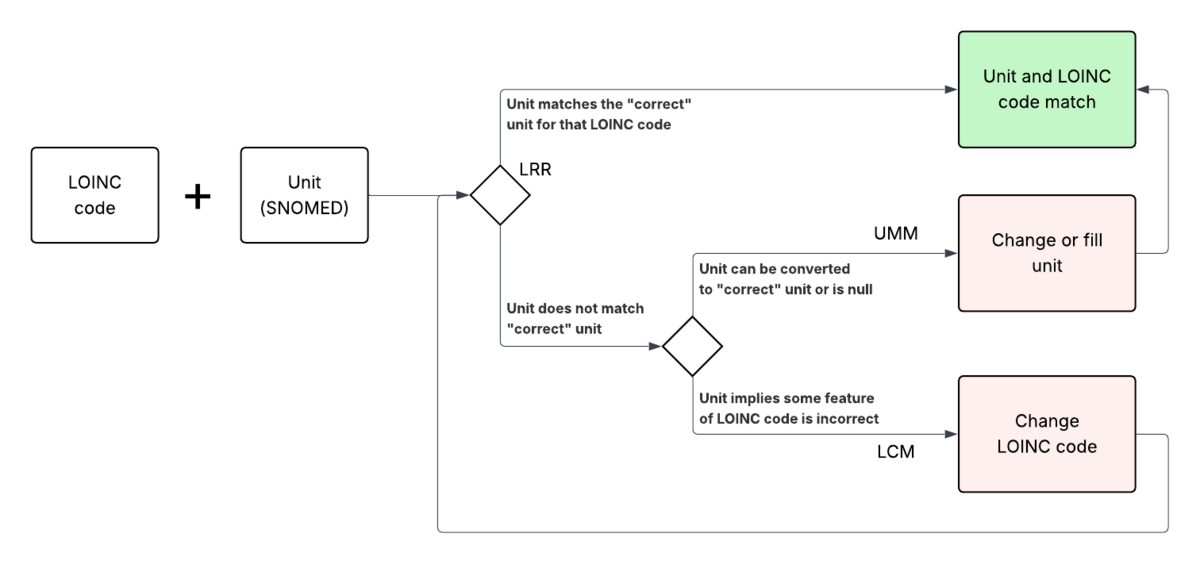
Framework logic and knowledge table use. LCM: Logical Observation Identifiers Names and Codes (LOINC) conversion map; LRR: laboratory reasonable range; SNOMED: Systematized Nomenclature of Medicine; UMM: unit multiplier map.

The framework logic is shown in Figure 1. Conformance to the selected “correct” unit is checked against the laboratory reasonable range (LRR) table entry. If the unit does not conform, the LCM provides a better LOINC code if one is known for the unit that is present in the record. In the second transformation, the unit is altered according to the unit multiplier map, normalized if it is a synonym, converted using a multiplier value, or swapped for the correct unit if it is a known error pattern or absent altogether. For example, the LOINC code 1920-8 (glucose [mass/volume] in blood) has the correct unit “mg/dL.” If a record with this LOINC code has the unit “mmol/L,” the LOINC code is changed using the LCM to 15074-8 (glucose [moles/volume] in blood). The process is only 2 steps, although not all records require both. Records may begin with LOINC code–unit conformance and skip the process altogether. Some require only LOINC conversion, some require only unit normalization, and many require LOINC conversion and then unit normalization.

Each of these transformations only proceeds when the record’s result value falls within the reasonable range associated with the LOINC code–unit pair that would appear in the transformed record. These ranges were selected using known correct data and designed to capture most possible values in that laboratory’s value distribution.

In rare instances, the system must select between 2 possible transformations for the same record. This might occur in the situation visible in rows 5 and 6 in Table 3 when trying to convert from g/dL to mg/dL. We identified a common error in many records in which a unit’s prefix was absent. A g/dL unit might be a simple data entry error at the source, or it may, in fact, require conversion by multiplying the result by 1000. The system uses the reasonable ranges to determine which of these 2 situations is more likely. If the reasonable ranges are not disparate enough to discern which transformation is required, proximity to the median of the target LOINC code–unit’s distribution is compared. The transformation resulting in a record closer to that median is selected. If the compared laboratory results have medians of 0, distance from the mean is used.

To evaluate the success of each transformation, the value distribution of the output for any individual transformation was compared with the value distribution of known correct data with that LOINC code–unit pair. If the distribution of the numeric result of the transformed data matched that of known correct data, it was considered successful. If the distribution did not match, the discrepancy was investigated. These investigations led to the discovery of common error patterns that could then be accounted for in the next iteration by adding to the 3 knowledge tables.

To summarize, the precedence of the rules described above is as follows:

1. LRR conformance check—if the LOINC code–unit pair conforms to the LRR table, no transformation is applied.
2. LOINC conversion (LCM)—if the unit is incompatible with the LOINC code, the framework first selects the appropriate LOINC code.
3. Unit normalization or conversion (unit multiplier map)—after the LOINC code is finalized, unit normalization or conversion is applied.
4. Range validation—a transformation is only accepted if the resulting value falls within the target LOINC code’s reasonable range.
5. Conflict resolution—when more than one unit transformation falls within the reasonable range, the system resolves this by checking proximity to the median of the known correct distribution or proximity to the mean when the distribution median is 0.

A few examples of LOINC code–unit incongruencies and framework processing are outlined in Multimedia Appendix 1.

Provenance is maintained within each transformed record in an attribute that acts as a ledger of each transformation that alters that record. This attribute is an array of objects that includes a descriptive string that details what features of the record were adjusted and what features were used to justify that adjustment for human readability. For computability, “object” includes the adjusted attribute names and preadjustment values for those attributes, ensuring transparency to downstream users and reversibility if necessary.

**Figure 2:**
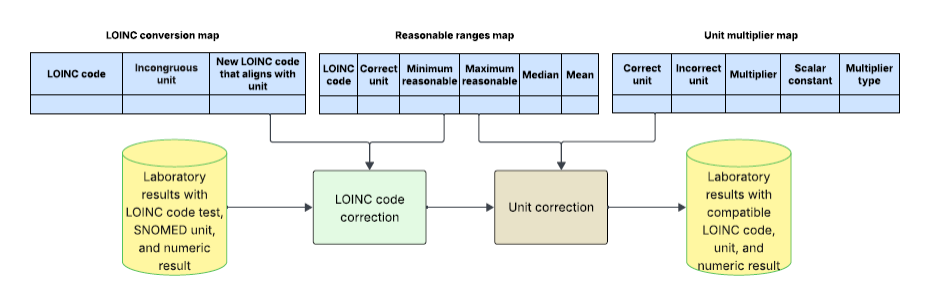
Summary of the framework. LOINC: Logical Observation Identifiers Names and Codes; SNOMED: Systematized Nomenclature of Medicine.

A summary of the steps for this framework is illustrated in Figure 2.

**Figure 3:**
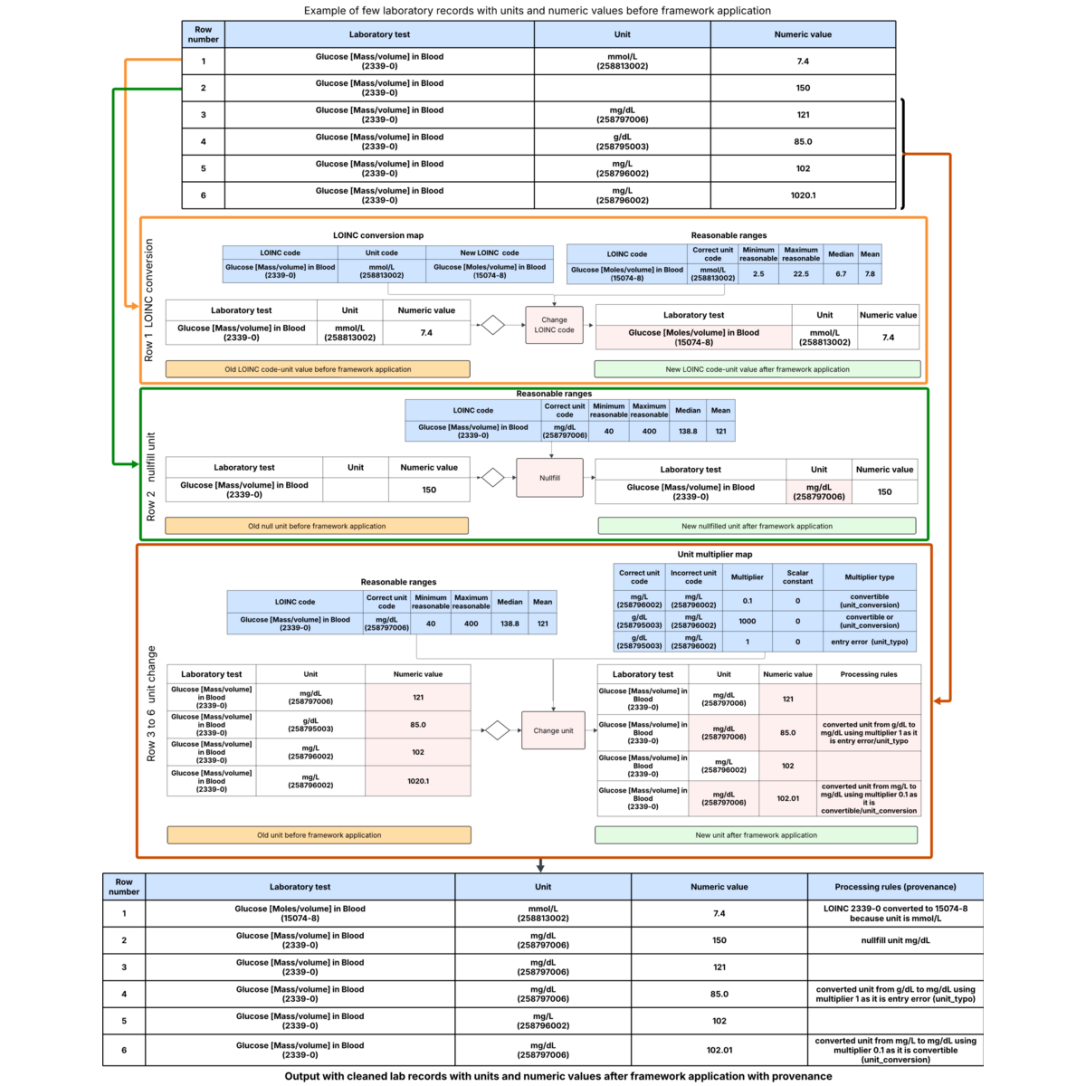
Example of Logical Observation Identifiers Names and Codes (LOINC) code–unit pairs before and after transformation.

Figure 3 shows an example of LOINC code–unit pairs before transformation, each step of the transformation, and the final output after transformation.

### Ethical Considerations

This study used retrospective, deidentified data collected for clinical purposes. All data were fully anonymized before analysis, and unique patient identifiers were removed. Analysis of deidentified secondary data does not constitute human subjects research and does not require formal review or approval by an institutional review board, and thus, no institutional review board approval number was sought. Because only deidentified data were used, informed consent from individual patients was not required. Privacy and confidentiality were maintained throughout the study in accordance with all applicable regulations.

## Results

### Dataset Collection: Overview and LOINC Coverage

All 4 sets of laboratory data underwent the LOINC code and unit correction process, and the proportion of records exhibiting each LOINC code–unit pattern was calculated both before and after the framework execution. A total of 428 LOINC codes were included in this study, chosen based on their prevalence in the ConcertAI database. Not all datasets contained all 428 LOINC codes. These LOINC codes accounted for 6.34 billion records in the ConcertAI database and 880 million, 2.98 billion, and 4.64 billion records in the source-specific datasets from sources 1, 2, and 3, respectively.

### System Design

#### Impact of Framework on Unit Correction

Before the application of the framework, 73.1% (4,634,610,173/6,337,101,453) records in the ConcertAI dataset with these LOINC codes had the correct unit as assigned by the LRR table. Following the application, this increased to 99.7% (6,322,375,200/6,341,230,213) of records. A similar trend was observed in each of the EHR-specific datasets: 78.5% (691,315,390/880,250,137) to 99.8% (879,626,472/881,157,852), 71.4% (2,132,455,936/2,985,465,124) to 99.8% (2,982,319,644/2,988,173,959), and 63.3% (2,936,710,502/4,640,432,294) to 99.6% (4,618,714,114/4,638,862,412) for sources 1, 2, and 3, respectively. The system increased the completion rate of units, defined as the proportion of records with nonmissing units, from 92.7% (5,879,071,858/6,341,230,213) to 99.8% (6,331,923,060/6,341,230,213) in the ConcertAI dataset and similarly in the other 3 datasets: 92.5% (814,867,241/881,157,852) to 100.0% (880,816,133/881,157,852), 94.4% (2,822,107,252/2,988,173,959) to 99.9% (2,986,624,027/2,988,173,959), and 91.7% (4,254,054,966/4,638,862,412) to 99.9% (4,632,935,919/4,638,862,412) for sources 1, 2, and 3, respectively. Because the framework assigns validated units to previously missing values, improvements in the correct unit rate reflect both corrections to misassigned units and the assignment of correct units to previously missing entries. As a result, the correct unit and completion rate metrics are related but not redundant: the correct unit rate captures accuracy among all populated units, whereas the completion rate captures overall presence of any unit.

#### LOINC-Level Quality Threshold

**Figure 4:**
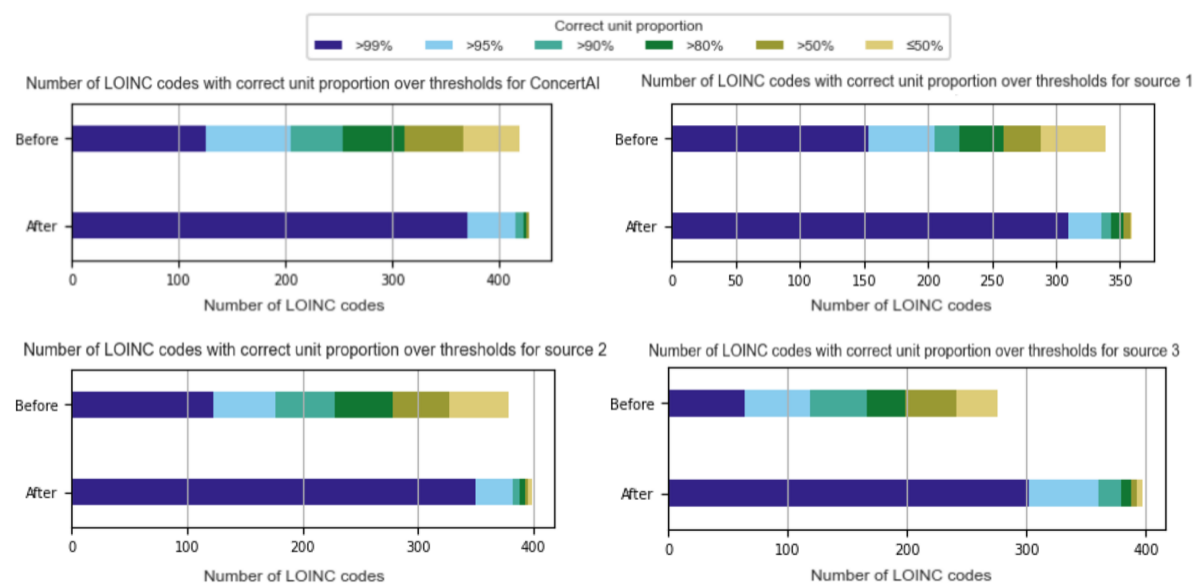
Number of Logical Observation Identifiers Names and Codes (LOINC)–coded records meeting unit correctness thresholds.

Not all LOINC codes start with the same degree of incorrectness. To evaluate the proportion of LOINC codes that reached specific data quality thresholds before and after framework application, records with each LOINC code were evaluated for unit correctness, and the number of LOINC codes passing certain unit correctness proportion thresholds was counted. These counts for each dataset are shown in Figure 4. Of the 428 LOINC codes, the number meeting the 99% threshold of unit correctness in the full ConcertAI dataset increased from 125 (29.2%) to 370 (86.4%). Similar improvements were observed across the 3 source datasets, which contained different total proportions of evaluated LOINC codes: 83.9% (359/428) of LOINC codes in source 1, a total of 93.2% (399/428) of LOINC codes in source 2, and 88.6% (379/428) of LOINC codes in source 3. In source 1, the proportion meeting the 99% threshold increased from 42.9% (154/359) to 86.4% (310/359); in source 2, it increased from 30.8% (123/399) to 87.7% (350/399); and in source 3, it increased from 16.9% (64/379) to 79.7% (302/379).

#### Illustrative Subset Analysis

**Figure 5:**
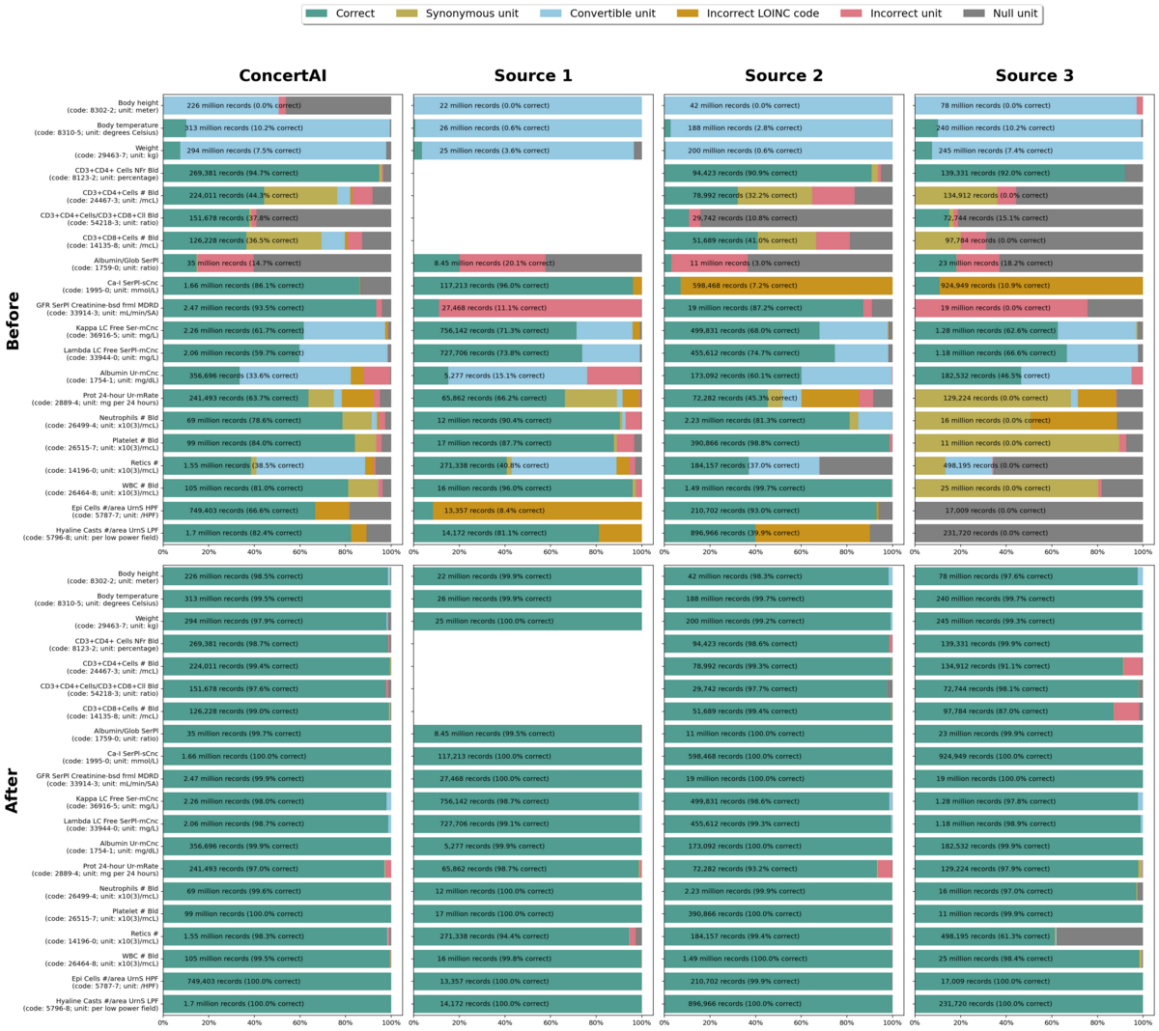
Error type proportions for specific Logical Observation Identifiers Names and Codes (LOINC) codes. GFR: glomerular filtration rate; HPF: high power field; MDRD: Modification of Diet in Renal Disease; SA: surface area; WBC: white blood cell.

To provide a more detailed view of the framework’s impact, Figure 5 presents a closer look at 20 specific LOINC codes. These codes were selected for their varied domains and types of incongruencies in the starting data.

Records with each of these 20 LOINC codes had their unit classified as correct, synonymous to correct, absent, or incorrect. Incorrect units were subclassified as incorrect and suggestive of a LOINC change, incorrect but convertible to the correct unit through a multiplier, or incorrect but not convertible. These unit categories represent the types of errors that this system is designed to correct. The proportions in each category were calculated before and after the framework application and are shown in Figure 5 for each system.

A detailed report showing the proportion of correctness for each LOINC code across all 4 datasets before and after the framework application is available in Multimedia Appendix 2.

## Discussion

### Principal Findings

The proposed normalization framework significantly improved LOINC code–unit congruence and unit absentia across all evaluated datasets, demonstrating generalizability to any clinical data system using standard medical terminologies for observation and unit representation. The method improved LOINC code–unit congruence overall in the ConcertAI laboratory dataset from 73.1% (4,634,610,173/6,337,101,453) to 99.7% (6,322,375,200/6,341,230,213), with similar trends in the other 3 data sources. The method and knowledge tables were developed using the ConcertAI data and applied to these independent datasets to demonstrate generalizability. To the best of our knowledge, this framework is the first of its kind to address 2 key gaps: the absence of a system-agnostic process for cleaning multisource laboratory data for secondary use [18] and an approach tailored to RWD. RWD, where data quality is both challenging [25] and critical to data usability, may lack source-specific semantics such as the local observation concept display name. This would impede the use of a standardization process designed to take advantage of those strings [18].

### Mechanism of Framework

The process, driven by 3 small, shareable knowledge tables, uses the observation concept (LOINC), the unit concept (SNOMED), and the numeric result as input. It first adjusts the LOINC code if the unit suggests a better LOINC code. It then normalizes the unit to a predetermined preferred unit for that LOINC code by filling null units, converting related units, or correcting units with overt errors. All transformations only proceed when the numeric result aligns with the predetermined distribution for the target LOINC code–unit pair. This “reasonable range” constraint addresses a shortcoming in existing proposed methodologies [19,21] that handle units without consideration of the numeric result. This range can be used to flag records that fall outside the reasonable distribution for exclusion from analysis or closer review.

### Impact on Data Quality Dimensions

Ensuring that laboratory values fall within a reasonable distribution contributes to accuracy, or the degree to which the data represent the true value of what is intended to be measured, and plausibility, 2 prominent dimensions [25] of RWD quality. The results demonstrate improvements across a number of established quality dimensions [25] other than accuracy and plausibility, including conformance, consistency, completeness, and provenance [25-29]. The technique primarily addresses conformance to the approved LOINC code–unit pair set by the LRR table and consistency of semantic representation regardless of data source. Completeness is improved by populating the unit when null. This contextually appropriate population of absent units has not been addressed in prior studies of similar techniques [19,21].

### Data Provenance

Data provenance is recognized as critical metadata for systems transmitting, storing, or using health data [30]. Records in RWD pipelines may undergo many transformations on their path to integration into a single dataset, and these transformations must all be tracked to enable error correction and improve trustworthiness with downstream consumers. Data provenance has been defined in many ways [31], and there are established specifications for recording provenance in health care data, such as the FHIR Provenance resource [32] based on the World Wide Web Consortium provenance specification [33]. Establishing the best method for provenance tracking within a system requires an assessment of the stakeholders for those data. The stakeholders for the transformations described in this paper were internal data analysts, informaticists, and quality assurance engineers. Provenance is maintained within each transformed record in an attribute that acts as a ledger of each transformation that altered that record. This attribute is an array of structures that include a descriptive, human-readable string detailing the transformation, as well as the altered attributes and preadjustment values that those attributes took. By consulting this array, transformations are fully reversible, original values are preserved, and a complete audit trail is available to support transparency and verification by downstream users.

### Clinical and Research Relevance

Laboratory data quality and usability are critical in systems handling oncology data, where these results are used to select and monitor therapies and assess disease severity and progression [34]. LOINC code–unit incongruence, unit absentia, and lack of unit standardization put an undue burden on any analyst or application attempting to use laboratory data for clinical decision support, retrospective studies, or clinical trial eligibility [35,36]. Improving unit completeness at the source is challenging because units are often inconsistently documented or transmitted in EHR systems [37,38]. Potential upstream improvements could include enforcing unit capture at the point of entry, not allowing free-text entry of units, ensuring consistent transmission in Health Level Seven and FHIR messages, or applying unit standardization at the health system level before data export. However, such approaches depend on local workflows and vendor configurations and cannot be guaranteed for multisource RWD. Consequently, postcodification harmonization frameworks such as the one described in this paper provide a practical and reproducible solution. The technique outlined in this paper presents a minimal set of logical steps to correct these problems within the pipeline of a multisourced data system handling RWD. Enforcing semantic consistency between LOINC and unit improves data usability [24] but can also foster better data exchange [39] between systems by reducing semantic differences. Ultimately, this framework ensures that laboratory data are both accurate and complete, enabling faster data-driven decisions that can enhance patient care, data exchange, clinical trials, and RWD analysis.

### Limitations and Future Work

The technique is presently limited by its capacity to correct the body system, which will reduce effectiveness for laboratory tests in which the potential body systems are relatively evenly distributed and share similar units when tested in either sample type. Information related to the body system can be present in the observation name but also in the panel name, the order name, discretely in another attribute in the laboratory result, or in other observations related to the result in question. Efforts are currently underway to evolve the process by incorporating panel or order-related information. The choice of SNOMED for unit may have been less ideal than UCUM [40,41], which is more flexible and more closely aligned with LOINC [42], which suggests a UCUM unit for some of its concepts. The FHIR [43] and Interoperability Standards Platform [44] also suggest that UCUM be used to represent units. However, the framework is easily adaptable by simply replacing the SNOMED codes used in the knowledge tables with UCUM codes without requiring any further changes in script. In addition, the steps described in this paper specifically address quantitative LOINC codes and require modification to be applicable to qualitative scale–type LOINC codes. Another limitation is that the creation of the knowledge tables requires clinical and terminologist expertise to recognize errors and prepare the tables, as well as a large dataset to prepare laboratory value distributions. This framework is designed for secondary use in RWD, where inconsistencies, missing values, and heterogeneous coding practices complicate analysis, and is not intended for integration into direct point-of-care clinical decision-making. Therefore, its use does not pose any direct safety risk to patients. The harmonization of LOINC code–unit combinations and the potential flagging of outlier values can influence downstream analyses such as cohort definitions or outlier detection. It enables data analysts to use laboratory data that would otherwise have required a significant time investment to clean. The primary aim of these corrections is to reduce inconsistencies and facilitate reliable data aggregation for secondary research purposes. Future work will focus on integrating the framework with emerging technologies such as artificial intelligence.

### Practical Implementation

To support practical implementation, we have released a starter kit supporting normalization of 146 LOINC codes from blood or serum, covering the “Hematology and cell counts” class and “Chemistry” glucose codes. It has 3 knowledge tables and an accompanying Python script available via a public GitHub repository [45]. The knowledge tables incorporate LOINC and SNOMED, which are maintained in multiple languages, allowing the tables to be used with datasets in different linguistic contexts. The numeric values in the tables are language independent and do not require conversion, supporting their generalizability to different languages. The Python script executing the rules takes FHIR Observation resources as input, and 6 example resources are provided.

Currently, the framework leverages SNOMED codes for units. However, the design is adaptable: implementers preferring UCUM codes can replace SNOMED codes with UCUM codes in the tables without any modification to the transformation script, allowing for seamless adoption of alternative unit standards. This flexibility ensures that the framework can be applied in diverse institutional or research contexts with minimal overhead.

A key component of the framework is its structured provenance tracking, which records all transformations applied to each laboratory record in a way that allows the original data to be reconstructed if needed. For each modification, information such as the applied rule, details of the transformation, which fields were changed, the original values, and the reference values used in the conversion is systematically captured. Collectively, these elements enable full traceability, providing a clear lineage from the transformed value back to the original measurement. This approach not only supports reproducibility and auditing but also ensures transparency, allowing researchers and data analysts to confidently interpret and validate the standardized data while retaining the ability to recover the source information.

By combining reproducible tables, adaptable unit standards, and robust provenance tracking, the framework provides a practical pathway for implementing consistent laboratory data harmonization in diverse settings while maintaining the transparency and traceability of each transformation.

### Conclusions

As the demand for multisourced RWD datasets grows, ensuring data quality and semantic consistency becomes increasingly vital. Persistent challenges regarding SI in laboratory data continue to limit the full potential of these data assets. Common issues include missing or incorrect units and misassigned LOINC codes, all of which can hinder integration and analysis. This framework addresses these challenges by systematically identifying and correcting discrepancies in quantitative laboratory data and has been shown to improve unit accuracy to above 99% for all evaluated datasets. It enables scalable, system-agnostic normalization of laboratory data, addressing critical gaps in several data quality dimensions. By improving laboratory data quality at the foundational level, it strengthens the reliability of RWD for data analysis, insight generation, and utility in software applications.

## Appendix

Multimedia Appendix 1 Detailed process for building the 3 knowledge tables described in system design, that is, the reasonable range map, unit multiplier map, and Logical Observation Identifiers Names and Codes conversion map.

Multimedia Appendix 2 Detailed report on the proportion of correct units before and after application of the framework, stratified by Logical Observation Identifiers Names and Codes code and by data source.

## Abbreviations

EHR: electronic health record
FHIR: Fast Healthcare Interoperability Resources
LCM: Logical Observation Identifiers Names and Codes conversion map
LOINC: Logical Observation Identifiers Names and Codes
LRR: laboratory reasonable range
RWD: real-world data
SI: semantic interoperability
SNOMED: Systematized Nomenclature of Medicine
UCUM: Unified Code for Units of Measure

## Data Availability

The datasets generated or analyzed during this study are not publicly available due to proprietary and licensing restrictions but may be available from ConcertAI, LLC, upon reasonable request and subject to applicable data use agreements and approval processes [46].
