# Supplementary material for: Harmonizing Logical Observation Identifiers Names and Codes (LOINC) Codes and Units in Real-World Oncology Data: Method Development and Evaluation
Source: JMIR Med Inform. 2026 Mar 9;14:e81254. doi: 10.2196/81254 (PMC13010070; doi:10.2196/81254)
Supplement: Multimedia Appendix 1 [file medinform_v14i1e81254_app1.docx]

## Section 1: Knowledge Tables Creation

The laboratory correction system employs three manually curated knowledge tables - Reasonable Ranges map, LOINC Conversion map, and Unit Multiplier map, developed through meticulous examination of the source dataset. These tables were constructed using only concept codes originating from the ConcertAI’s codification process, chosen due to their robust provenance ensuring maximal accuracy. The data was restricted to records with identifiable numeric results. Aggregate measures, including incidence, mean, median, minimum value, maximum value, and percentile distributions (0.5th, 2.5th, 5th, 16th, 50th, 84th, 95th, 97.5th, and 99.5th), were calculated for all LOINC code-unit code pairings. Subsequently, LOINC codes were rank ordered by incidence and value for individual analysis. The system is currently active for 428 LOINC codes.

Creation of the knowledge tables involved identifying all patterns in the input data and configuring rules, represented as rows in the tables, to address each nonconformant LOINC-unit pattern. Each pattern found could lead to further investigation of where these inconsistencies originate. Typically, it involved an observation string that was insufficient to correctly choose all 6 axes of LOINC, e.g., ‘white blood cells’ does not allow you to distinguish between the body systems of urine or blood. Sometimes these investigations highlighted coding errors or suggested coding improvements that could be corrected upstream. The process of knowledge table creation itself was an exercise in data quality improvement and provided a framework for lab data evaluation.

The starter kit GitHub repository provides the computable script and csv versions of these tables: https://github.com/PrecisionHealthIntelligence/loinc_unit_harmonization

### A. Reasonable Range Map

This table defines metadata about a single LOINC code including its assigned correct unit of measure, a minimum and maximum value that the lab can reasonably take, as well as the mean and median of records with that LOINC code and unit. For each LOINC code, a correct unit code was selected, using the example_unit supplied in the official LOINC distribution. These units were mapped to the corresponding SNOMED codes. For LOINC codes that listed more than one example unit, we examined the empirical distribution of units observed in our dataset and selected the unit with the highest frequency. These LOINC and “correct unit” pairings were then used to determine a reasonable range for each LOINC code. Minimum and maximum reasonable values for each LOINC code were defined by estimating the empirical distribution of the data through quantiles of 0.005, 0.025, 0.16, 0.5 (median), 0.84, 0.975, and 0.995. The range was set at or near the upper and lower of those quantiles, providing broad yet data-anchored thresholds for plausibility checking. This range will be used to define when we can safely perform a correction to the LOINC code or unit in cases where the pairing is considered incorrect. The selected reasonable range subsumes the lab’s reference range and captures the majority of results in our system for that LOINC code and unit. It accounts for the possibility that some labs may report greatly elevated, even supraphysiological, values in diseased individuals.

The correct unit, the reasonable range, as well as mean and median for each LOINC code where the unit is known to be correct, together comprise the table. This structure makes it computable for the framework.

This table contains the following columns:

loinc_code (String): LOINC identifier representing the standardized laboratory test (pkey).

loinc_name (String): LOINC standardized name for the laboratory test.

unit_code (String): Standard code representing the unit of measure for the laboratory test.

unit_name (String): Standard name for the unit of measure associated with the laboratory test.

min_reasonable (Numeric): Lower bound of the reasonable range for the LOINC-unit pair, derived using five standard deviations. This threshold includes the lab’s reference range while allowing for abnormally low results when clinically plausible.

max_reasonable (Numeric): Upper bound of the reasonable range for the LOINC-unit pair, derived using five standard deviations. This threshold encompasses the lab’s reference range and accommodates cases where labs may report markedly elevated or supraphysiological values when clinically plausible.

mean (Numeric): Mean of numeric value for the LOINC-unit pair.

median (Numeric): Median of numeric value for the LOINC-unit pair, representing the 50th percentile.

An example of reasonable ranges is shown in Table 1.

**Table 1: Lab reasonable ranges.**

| **loinc_code** | **loinc_name** | **unit_code** | **unit_name** | **min_reasonable** | **max-reasonable** | **median** | **mean** |
| --- | --- | --- | --- | --- | --- | --- | --- |
| 26515-7 | Platelets [#/volume] in Blood | 1287856009 | x10(3)/mcL | 1 | 800 | 223.5 | 215.0 |
| 2339-0 | Glucose [Mass/volume] in Blood | 258797006 | mg/dL | 40 | 400 | 138.8 | 121 |
| 11050-2 | Kappa light chains [Mass/volume] in Serum or Plasma | 258796002 | mg/L | 0.1 | 5000 | 68.7 | 25.9 |

### B. LOINC Conversion Map

This table elates a LOINC concept and unit concept that are incongruous to a new LOINC code that is compatible with the unit of measure. The LOINC-unit pairs that are incongruent were utilized to construct this. When a unit was determined to be inconsistent with the LOINC code's attributes (like property), a record was added to the LOINC conversion map, specifying that incorrect unit. The correct LOINC code was identified by searching for codes with similar attributes that aligned with the information present in the unit. Typically, the unit's information suggested a different LOINC 'property' attribute, but it could also indicate a similar yet distinct component, a different time aspect, a different method, or a different body system. These incorrect LOINC-unit pairings, along with their corresponding corrected LOINC codes, constitute this knowledge table.

This table contains the following columns:

old_loinc_code (String): LOINC code requiring conversion because its unit pairing does not reflect the correct LOINC property.

old_loinc_display (String): Human-readable name or display text for the LOINC code requiring conversion.

old_loinc_system (String): Standard terminology system for the standard code requiring conversion i.e., LOINC.

unit_code (String): Standard code representing the unit of measure for the laboratory test.

unit_display (String): Standard name for the unit of measure associated with the laboratory test.

unit_system (String): Standard terminology system for the unit code (e.g., SNOMED or UCUM)

new_loinc_code (String): LOINC code to which the original LOINC can get converted to.

new_loinc_display (String): Human-readable name or display text for the LOINC code to which the original code can be converted to.

new_loinc_system (String): Standard terminology system for the corrected standard code i.e., LOINC.

The primary key is a combination of old_loinc_code and unit_code. Table 2 provides examples of this table.

**Table 2: LOINC conversion map.**

| **old_loinc_code** | **old_loinc_display** | **old_loinc_system** | **unit_code** | **unit_ display** | **unit_system** | **new_loinc_code** | **new_loinc_display** | **new_loinc_system** |
| --- | --- | --- | --- | --- | --- | --- | --- | --- |
| 26515-7 | Platelets [#/volume] in Blood | LOINC | 258775009 | fL | SNOMED | 28542-9 | Platelet [Entitic mean volume] in Blood | LOINC |
| 2339-0 | Glucose [Mass/volume] in Blood | LOINC | 258865000 | mEq/L | SNOMED | 15074-8 | Glucose [Moles/volume] in Blood | LOINC |
| 2339-0 | Glucose [Mass/volume] in Blood | LOINC | 258813002 | mmol/L | SNOMED | 15074-8 | Glucose [Moles/volume] in Blood | LOINC |
| 11050-2 | Kappa light chains [Mass/volume] in Serum or Plasma | LOINC | 118582008 | Percent | SNOMED | 17096-9 | Kappa lymphocytes/Lymphocytes in Blood | LOINC |

### C. Unit Multiplier Map

This table relates a correct unit to a synonymous, convertible, or incorrect unit along with a multiplier value, an additive scalar value, and a description of the relationship between the two units, e.g., incorrect, synonym, or convertible. These units that need conversion are the units that are still reflective of the corresponding LOINC property but need to be converted to one correct standardized unit, where applicable. These unit conversions are created from the units that can be reliably converted to the correct unit as defined in lab_reasonable_ranges table by multiplying the value by a constant factor.

For synonymous and null unit conversions, we assigned a multiplier of 1. The units that need an actual conversion with a multiplier are given a non-1 multiplier.

When an erroneous unit appeared frequently enough in the system, its value distribution was compared against the distribution for the correct unit in the same lab. If the distributions matched, the unit was treated as a ‘Known error’ incorrect unit (e.g., Table 3, Row 4, 6, 9). With this, we now populate the Unit Multiplier map with the incorrect unit paired with its corresponding correct units and the multiplier of 1. These ‘known error’ types include missing denominators, missing numerators, or occasionally a missing scaling factor like the character µ (e.g., Table 3, Row 8). The complete absence of a unit is also frequently encountered. In cases where the value distribution of a LOINC code paired with a null unit aligns with the correct unit's distribution, the absent unit is imputed using the unit multiplier map (e.g., Table 3, Row 1, 10).

It is also possible that a null or incorrect unit necessitates conversion using a multiplier to align with the laboratory's correct value distribution. These two possibilities, a known error that either requires or does not require conversion, can occur concurrently within a LOINC-"incorrect unit" pairing. This phenomenon manifests as a bimodal distribution, where the first mode corresponds to the correct distribution's peak, and the second mode requires a multiplier for alignment. To accommodate this pattern, two rows are added to the unit multiplier map with the same unit pair but different multipliers (e.g., Table 3, Row 3, 4 for converting incorrect unit cells/uL to correct unit x10(3)/mcL. Similarly, Row 5, 6 to convert to correct unit mg/dL). When a unit correction was performed on records with this incorrect unit, the multiplier to apply was selected based on whether the converted value fell within the reasonable range. If both multipliers result in a value within the reasonable range, the proximity to the lab’s “correct” median was used to determine the applied multiplier. If the lab’s median was 0, then the mean was used in place of the median.

**Table 3: Unit multiplier map.**

| **incorrect_unit_code** | **incorrect_unit_name** | **correct_unit_code** | **correct_unit_name** | **multilier** | **sclar** | **multiplier_type** |
| --- | --- | --- | --- | --- | --- | --- |
| Null_code |  | 1287856009 | x10(3)/mcL | 1 | 0 | Unit absent |
| 277288007 | 10*9/L | 1287856009 | x10(3)/mcL | 1 | 0 | Synonym |
| 258878000 | cells/uL | 1287856009 | x10(3)/mcL | 0.001 | 0 | Convertible |
| 258878000 | cells/uL | 1287856009 | x10(3)/mcL | 1 | 0 | Known error |
| 258795003 | g/dL | 258797006 | mg/dL | 1000 | 0 | Convertible |
| 258795003 | g/dL | 258797006 | mg/dL | 1 | 0 | Known error |
| 258794004 | g/L | 258797006 | mg/dL | 1 | 0 | Convertible |
| 258770004 | mg | 258797006 | mg/dL | 1 | 0 | Missing denominator |
| 258798001 | mg/mL | 258797006 | mg/dL | 1 | 0 | Known error |
| Null_code |  | 258797006 | mg/dL | 1 | 0 | Unit absent |

The following are columns in Unit multiplier map (pkey is combination of incorrect_unit_code, correct_unit_code and multiplier_type)

incorrect_unit_code (String): Unit code that requires conversion.

incorrect_unit_name (String): Human-readable name of the unit that requires conversion.

correct_unit_code (String): Standard unit code to which the incorrect unit can get converted to.

correct_unit_name (String): Human-readable name of the standard unit to which the incorrect unit can get converted.

multiplier (Numeric): Factor by which the original numeric value should be multiplied during conversion to the new corrected unit.

scalar_constant (Numeric): Number to be added or subtracted from the original numeric value during conversion to the new corrected unit.

multiplier_type (String): Type or nature of the conversion applied, e.g., “typo_correction,” “synonym,” or other adjustment method.

These tables are computable structures and are now ready to be used in the workflow of the proposed methodology. Figure 1 below summarizes the workflow.

**Figure 1: Summary of the workflow.**


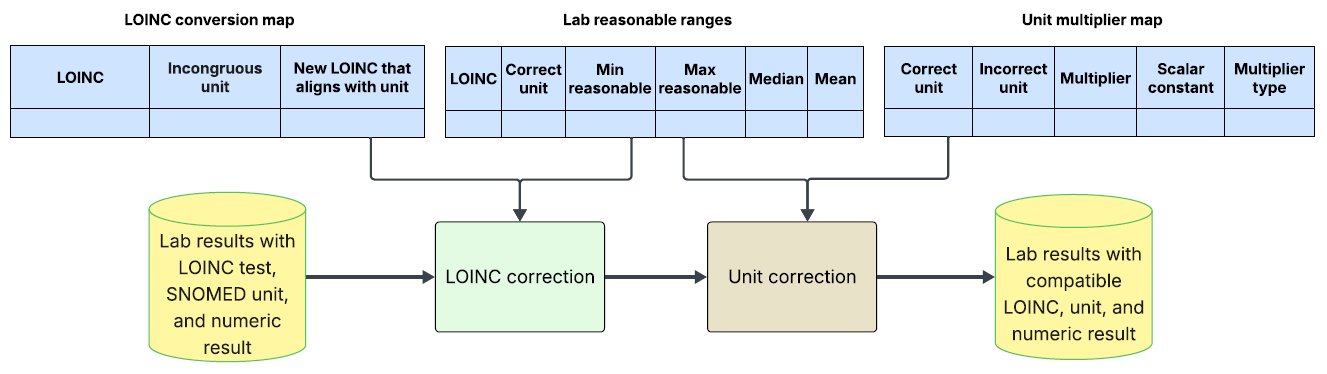


## Section 2: Example of LOINC-unit incongruencies

Table 4 outlines the logic using a few examples of LOINC-unit incongruencies for Glucose [Mass/volume] in Blood, which has a correct unit of mg/dL and a reasonable range of 40 to 400 mg/dL. In the first row, the unit mmol/L conflicts with the LOINC, so the LOINC is changed to match the unit. The second and third rows both represent the LOINC, unit and value of the same record but demonstrate two different transformations that are attempted on the record. In the second row, a unit conversion is attempted with a multiplier of 1000. In row 3, the unit conversion does not use a multiplier as it assumes the record to have a data entry error in the unit. The unit conversion proceeds for this record with the logic on row 3 and not row 2, correcting the unit from g/dL to mg/dL as 85 (row 3) falls within the reasonable range of 40 to 400, whereas 85,000 (row 2) does not. The fourth row has a null unit which is populated with mg/dL, after validating that the value falls within the reasonable range. In the fifth row, a unit conversion (mg/L to mg/dL) attempts to proceed with value multiplier of 0.1, resulting in a value of 10.2. Since 10.2 is not within the reasonable range, this conversion does not occur, and the original record remains unaltered by the process.

**Table 4**: Framework logic examples.

| LOINC | Value | Unit | Problem | Action | Validate against reasonable range |
| --- | --- | --- | --- | --- | --- |
| Glucose [Mass/volume] in Blood (2339-0) | 7.4 | mmol/L (258813002) | Unit and LOINC in conflict | Convert LOINC to Glucose [Moles/volume] in Blood | Is value 7.4 between 2.5 and 22.5? Yes |
| Glucose [Mass/volume] in Blood (2339-0) | 85 | g/dL (258795003) | Unit does not match correct unit | Convert unit to mg/dL and multiply value by 1000 | Is value 85,000 between 40 and 400? No |
| Glucose [Mass/volume] in Blood (2339-0) | 85 | g/dL (258795003) | Unit does not match correct unit | Convert unit to mg/dL but assume unit has data entry error and does not require value multiplier | Is value 85 between 40 and 400? Yes |
| Glucose [Mass/volume] in Blood (2339-0) | 150 |  | Unit is null | Fill in the null unit with mg/dL and does not require value multiplier | Is value 150 between 40 and 400? Yes |
| Glucose [Mass/volume] in Blood (2339-0) | 102 | mg/L (258796002) | Unit does not match correct unit | Convert unit to mg/dL and multiply value by 0.1 | Is value 10.2 between 40 and 400? No |
